# Rapidly-migrating and internally-generated knickpoints can control submarine channel evolution

**DOI:** 10.1038/s41467-020-16861-x

**Published:** 2020-06-19

**Authors:** Maarten S. Heijnen, Michael A. Clare, Matthieu J. B. Cartigny, Peter J. Talling, Sophie Hage, D. Gwyn Lintern, Cooper Stacey, Daniel R. Parsons, Stephen M. Simmons, Ye Chen, Esther J. Sumner, Justin K. Dix, John E. Hughes Clarke

**Affiliations:** 1 National Oceanography Centre, European Way, Southampton, SO14 3ZH UK; 2 Ocean and Earth Sciences, National Oceanography Centre, University of Southampton, European Way, Southampton, SO14 3ZH UK; 3 Departments of Geography and Earth Sciences, University of Durham, South Road, Durham, DH1 3LE UK; 4 Natural Resources Canada, Geological Survey of Canada, Box 6000, 9860 West Saanich Road, Sidney, BC Canada; 5 Energy and Environment Institute, University of Hull, Cottingham Road, Hull, HU6 7RX UK; 6 Earth Sciences, Center for Coastal & Ocean Mapping, University of New Hampshire, 24 Colovos Road, Durham, NC USA; 7 Present Address: Department of Geoscience, University of Calgary, T2N 1N4, Calgary, Alberta Canada

**Keywords:** Geomorphology, Sedimentology

## Abstract

Submarine channels are the primary conduits for terrestrial sediment, organic carbon, and pollutant transport to the deep sea. Submarine channels are far more difficult to monitor than rivers, and thus less well understood. Here we present 9 years of time-lapse mapping of an active submarine channel along its full length in Bute Inlet, Canada. Past studies suggested that meander-bend migration, levee-deposition, or migration of (supercritical-flow) bedforms controls the evolution of submarine channels. We show for the first time how rapid (100–450 m/year) upstream migration of 5-to-30 m high knickpoints can control submarine channel evolution. Knickpoint migration-related changes include deep (>25 m) erosion, and lateral migration of the channel. Knickpoints in rivers are created by external factors, such as tectonics, or base-level change. However, the knickpoints in Bute Inlet appear internally generated. Similar knickpoints are found in several submarine channels worldwide, and are thus globally important for how channels operate.

## Abstract (web summary)

The authors analyse 9 years of time-lapse surveys in Bute Inlet, British Columbia (CA), to show how an active submarine channel evolves. They show how channel evolution is controlled by fast upstream-migration of steep knickpoints, which are similar to waterfalls in rivers.

## Introduction

Seafloor sediment flows called turbidity currents transport globally important volumes of sediment, and form some of the deepest canyons, longest channels, and largest sediment accumulations on Earth^1–3^. These widespread underwater channel systems can extend for tens to thousands of kilometres offshore, and their dimensions may rival or even exceed those of terrestrial river systems^4,5^. Turbidity currents that flush submarine channels can be very powerful (reaching velocities of 20 m/s), and they pose a serious hazard to seafloor infrastructure, which includes telecommunication cables that carry >95% of global data traffic^6–8^. Furthermore, sediment, organic carbon, nutrients, and pollutants that are transported via submarine channels, influence deep marine ecosystems and climate on long time scales^9–11^, while ancient channel deposits can form reservoirs and source rocks for hydrocarbon production^12,13^, and act as an archive for the Earth’s history^14,15^.

Despite the global occurrence and importance of submarine channel systems, there are very few detailed time-lapse seabed surveys showing directly how channels evolve and change through time. Channels can evolve over different timescales, ranging up to “channel life cycles”, encompassing channel inception, maintenance and abandonment, which can span over geological times^16^. Here we describe channel evolution during its active (maintenance) stage.

We are aware of 17 locations where multiple bathymetric surveys of the modern seafloor have provided time-lapse information on how active channels evolve (Supplementary Table 1). These studies typically involve two surveys, cover periods of less than 5 years, do not cover the full extent of a system from source to sink, or capture relatively small delta-front systems. The highest resolution time-lapse study of a full-length system is from the 1–2 km long delta-front channels on the Squamish Delta, but this system is re-establishing itself after a man-made river diversion^17,18^. This lack of time-lapse studies is in stark contrast to the very large number of time-lapse studies of how river channels evolve, which benefit from abundant airborne lidar, aerial photographs, and satellite images^19^. There is a compelling need for detailed time-lapse studies to understand how submarine channels evolve.

This lack of time-lapse data from full-length systems ensures that previous studies of submarine channel evolution were mainly based on physical laboratory-scale modelling, numerical models, geophysical (seismic) data, outcrop studies, comparisons to rivers, and non-time-lapse seafloor mapping^20–24^. These studies have advanced considerably our understanding of how submarine channels work. However, laboratory models suffer from scaling issues^21^, and numerical models have to make assumptions that are often poorly validated against full-scale field data. Seismic data and rock outcrops only capture the end result of channel evolution, rather than a time series of how the channel evolved in response to certain environmental conditions. Intervals dominated by erosion are especially difficult to reconstruct using seismic data or rock outcrops. The resolution of seismic data is often insufficient to resolve small features within channels. Rock outcrops also lack detailed chronological data for quantifying rates of short-term processes, and may not give a full three-dimensional perspective^25^.

Despite these limitations, previous work has proposed three main processes that might control the evolution of submarine channels. First, it has been proposed that submarine channels evolve in a broadly comparable way to meandering rivers, via gradual outer-bend erosion and inner-bend deposition, and meander bend cut-off^5,23,26^. Bend migration and cut-off is primarily driven by cross-channel (secondary) flow and has long been known to be a dominant control on how rivers evolve^27–30^, and also occurs in submarine channels^31^. However, submarine channels have been suggested to differ in key regards from rivers^32^. Second, deposition of flanking levees may control channel evolution through confinement of turbidity currents; hence fixing the system in place.^33^. Third, it has been suggested that turbidity currents have a greater tendency than rivers to be Froude-supercritical (i.e. exist in a thin and fast state)^34^. Flow instabilities called cyclic-steps can characterise these supercritical turbidity currents, causing repeated hydraulic jumps. Crescent-shaped bedforms and repeated seabed scours are common expressions of these cyclic steps, which previous authors propose play a key role in submarine channel initiation, evolution, and deposit geometries^16,17,22,35–39^.

Another possible major control on submarine channel evolution could be the rapid migration of internally generated knickpoints. Knickpoints are steep steps in channel gradient that migrate upstream via erosion^40,41^, and they are common in rivers^42–44^. The knickpoint’s steep face enhances the erosive potential of flow, causing the knickpoint to migrate upstream. Sediment flux downstream of the knickpoint increases as a result of this enhanced erosion, causing more deposition on the next lower gradient section downstream^45^. Previous studies have shown that knickpoints are common in submarine (and sublacustrine) channels in various settings worldwide (Supplementary Table 2). A recent study of the head of a submarine canyon has shown that knickpoints can migrate up to 600 m/year, and leave a distinct pattern of erosion and deposition in the channel^46^.

**Fig. 1:**
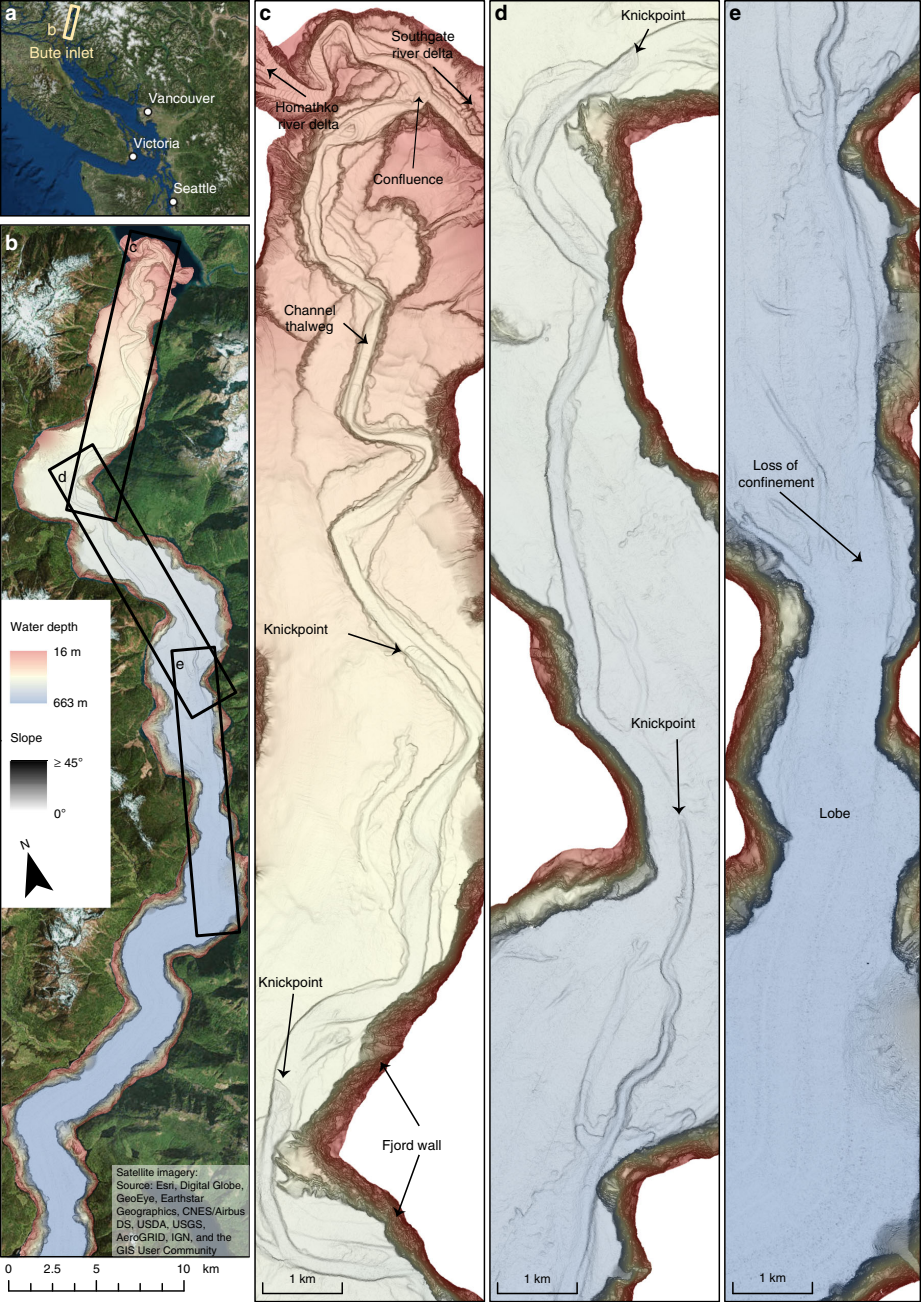
Overview of the submarine channel system in Bute Inlet. **a** Location of Bute Inlet in British Columbia, Canada. **b** Map of Bute Inlet showing the location of more detailed images shown in panels **c** to **e**. Bathymetric surveys are presented here as maps of seabed gradient, which optimally visualize small and steep topographical features, such as knickpoints. Seabed gradient maps are then overlain by a transparent bathymetry map. **c**-**e** Detailed maps of the 40 km long submarine channel within Bute Inlet, showing the location of river deltas, knickpoints and lobe beyond the channel mouth.

Here we present the most detailed time-lapse mapping yet for an active submarine channel, over its full length of ~40 km, to understand the role of migrating knickpoints in submarine channel evolution. These data comprise five bathymetric surveys over 9 years (2008–2016) in Bute Inlet, British Columbia, Canada (Fig. 1). These data allow us to document how a submarine channel evolves along its full length, for almost a decade. Our initial aim is to understand what factors can control the evolution of submarine channels. These time-lapse surveys show that the evolution of this submarine channel is dominated by rapidly migrating knickpoints. Our second aim is therefore to understand what causes these very fast-moving knickpoints. Our third aim is to understand the implications of these rapidly migrating knickpoints for submarine channel-bend evolution, and deposits preserved within channels. We provide new generalised models for both bend evolution and channel deposits. We conclude by showing that similar submarine knickpoints occur in many locations, and may thus have widespread importance for how submarine channels work, and how their deposits form.

## Results and discussion

### Geographical setting

Bute Inlet is located in British Columbia, West Canada (Fig. 1a). The head of this fjord is fed by the Homathko River and Southgate River, which are responsible for, respectively, 80% and 15% of the freshwater input in the system, with the remaining 5% from smaller rivers on the side of the fjord^47^. The rivers are mainly fed by glacial meltwater, with much higher discharges in summer. The Homathko River has an average summer discharge of 600 m^3^/s, with maxima above 1000 m^3^/s, while winter discharges are typically below 100 m^3^/s. It has been estimated that these rivers supply 1.6 × 10^6^ m^3^ of sediment to the fjord each year^47^. A ~40 km long submarine channel is present on the floor of Bute Inlet, and it originates at the pro-deltas of the two main rivers^48,49^. The channel is 35 m deep in the most upstream part of the system, and its depth decreases gradually downstream towards the depositional area (terminal lobe), beyond the channel termination at 620 m water depth^50^ (Supplementary Fig. 1).

The floor of the channel comprises sand, whilst the surrounding fjord is dominated by mud^48,50^. Turbidity currents occur frequently along the upper channel, with over 10 flows a year, which occur coincident with periods of higher river discharge in the spring and summer^49–51^. More recent and higher resolution bathymetric surveys demonstrated that the submarine channel in the Bute Inlet system is strongly altered by these turbidity currents, with 25% of the channel having changed elevation by 5 m or more within 3 years^52^ and showed active upstream migrating knickpoints^53^. Here we analyse a longer time series over a more extensive area of the submarine channel.

### Bathymetric changes and knickpoints

**Fig. 2:**
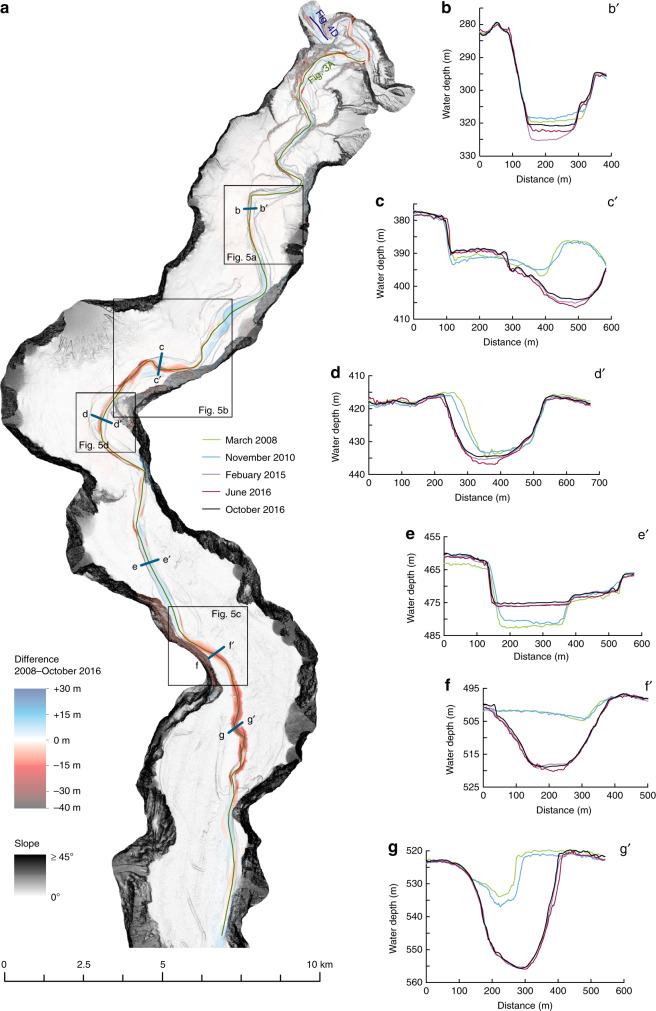
Changes in the submarine channel in Bute Inlet. **a** Map of changes in seabed elevation between March 2008 and October 2016, overlaying a seabed gradient map. Note the alternations of deposition and erosion along the channel. **b**-**g** Changes in seabed elevation at a series of cross-sections. Locations are shown in panel **a**. 10x vertical exaggeration. **b** The channel gradually fills except during knickpoint migration between 2010 and 2015, when previous deposits are eroded. **c** Lateral migration of channel thalweg as a result of knickpoint migration. Note how the channel floor in 2008-2010 becomes a terrace from 2015 onwards. **d** Section showing largest observed amount of outer-bend erosion away from migrating knickpoints. **e** Progressive filling of a channel in a depositional area. **f** Knickpoint migration creates a channel, where the channel was previously shallow and poorly-defined. **g** Cross section at a location affected by both outer-bend erosion and knickpoint migration.

**Fig. 3:**
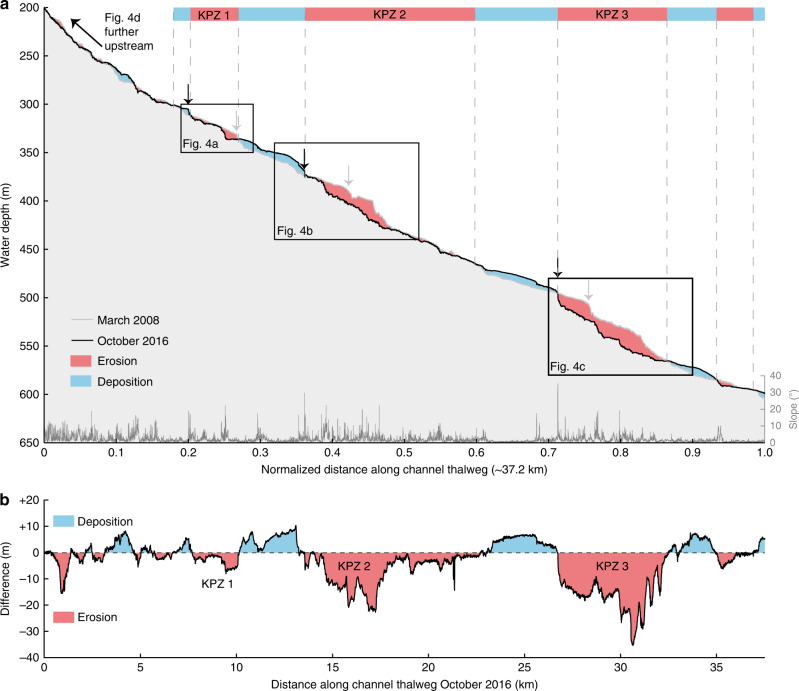
Changes along the channel profile in Bute Inlet. Location is shown in Fig. 2a. KPZ = knickpoint-zone **a** Bathymetric profiles along the channel thalweg in 2008 and October 2016. 50x vertical exaggeration. The position of the channel shifts as the channel evolves, so profiles were constructed along the position of the thalweg in that survey. Profiles were then normalised to allow comparison. Slope was generated using the survey from October 2016. Note the downstream alternation of deposition (blue) and erosion (red). Three main erosional areas (knickpoint-zones) are bounded at their upstream end by steep steps (frontal-knickpoint) in the channel profile. Additional smaller knickpoints are often present within wider knickpoint-zones. Proximal erosion upstream of knickpoint-zone 1 is due to lateral migration of the channel, unrelated to knickpoint migration. **b** Difference in channel elevations between March 2008 and October 2016 along the channel thalweg. Migration of three knickpoint zones (KPZ 1 to 3) produces erosional areas.

**Fig. 4:**
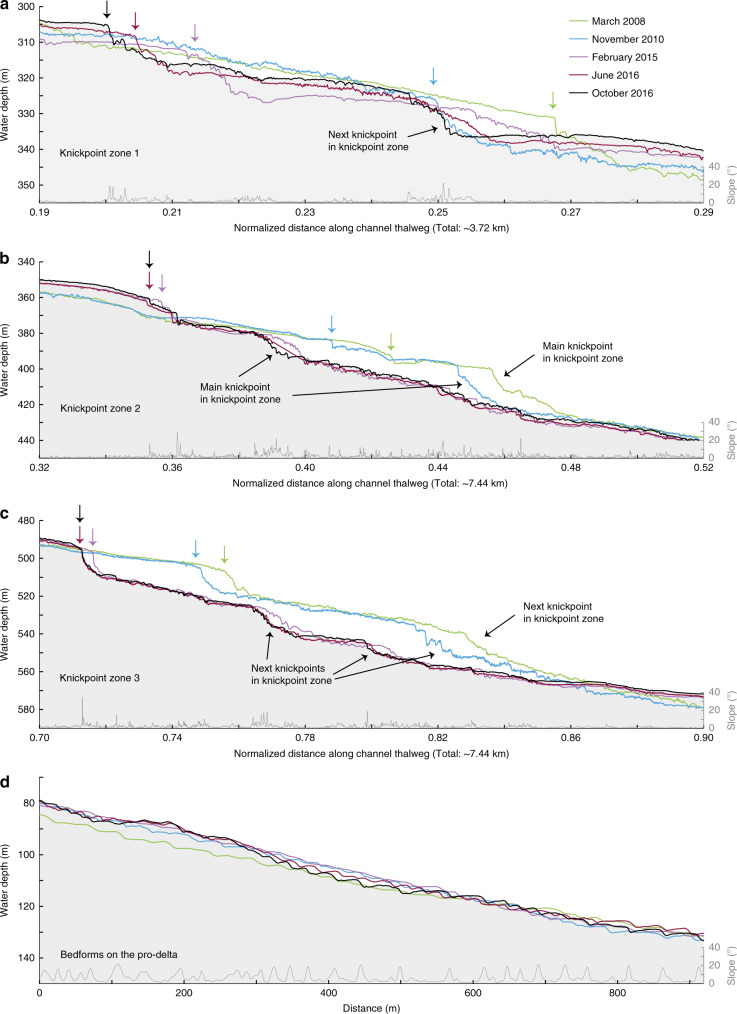
Time-lapse evolution of sections of the submarine channel profile. **a**-**c** Detailed time-lapse changes in profiles across knickpoint-zones 1, 2 and 3, whose locations are indicated in Fig. 3a. 20x vertical exaggeration. Slope was generated using the survey from October 2016.Arrows indicate the position of the frontal frontal-knickpoint in each survey. **d** Profile along the shallow-water pro-delta channel, as indicated in Fig. 2a, where crescentic shape bedforms dominate and no knickpoints are present. 5x vertical exaggeration. Note the relatively small amount of bathymetric change, when compared to the three knickpoint zones.

**Fig. 5:**
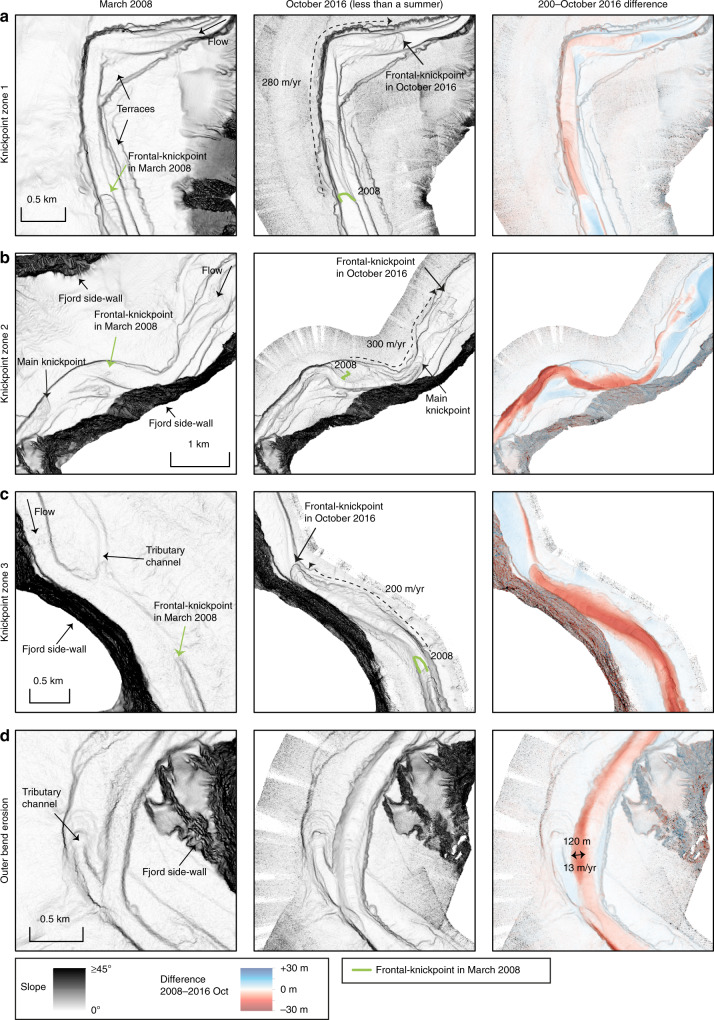
Detailed maps showing overall change in the three knickpoint zones and in a meander bend. Locations shown in Fig. 2a. Average migration rate of the frontal-knickpoint is indicated. **a** Bathyemtric change in knickpoint-zone 1. **b** Bathymetric change in knickpoint-zone 2. **c** Bathymetric change in knickpoint-zone 3. Knickpoint migration creates a channel, where previously no well-developed channel existed. **d** Largest erosion in an outer-bed not affected by knickpoint migration.

**Fig. 6:**
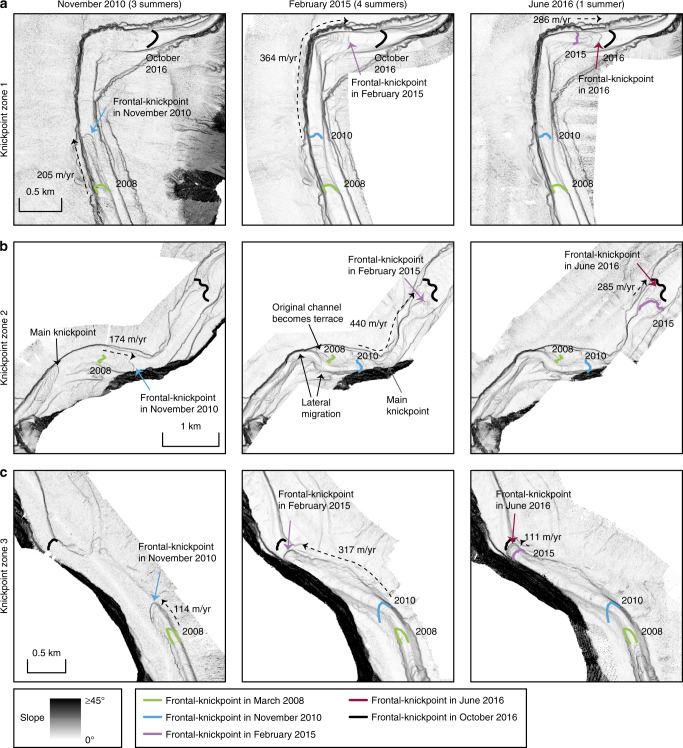
Detailed time-lapse maps in the three knickpoint zones. Locations shown in Fig. 2a. Time steps from March 2008 and October 2016 are shown in Fig. 5. **a** Time-lapse evolution of knickpoint-zone 1. **b** Time-lapse evolution of knickpoint-zone 2. Knickpoints migrate outside of the original channel, causing lateral migration of the channel in some parts. The original channel floor locally becomes a terrace. The resulting channel is narrower and more sinuous. **c** Time lapse evolution of knickpoint-zone 3. Knickpoint migration creates a channel where previously no well-developed channel existed.

A difference map captures bathymetric changes in the channel for the entire study period between March 2008 and October 2016 (Fig. 2a). It covers the full length of the channel, and the area immediately beyond the channel termination (start of the terminal lobe). The channel floor is characterised by alternating areas of erosion and deposition (Fig. 2a), a pattern that is repeated three times along the channel (Figs. 2a and 3a). The three main erosional areas are bounded at their upstream sides by a steep (up to ~30°) face that is 5–30 m high. Similar steep steps are found within each erosional area. These steep steps are called knickpoints, and here we refer to erosional areas that consist of several knickpoints as knickpoint-zones. Knickpoints bounding the knickpoint-zone at its upstream side are termed frontal-knickpoints. Repeat surveys show that frontal-knickpoints and associated knickpoint-zones migrate upstream between each pair of surveys (Figs. 3a, 4a–c, 5a–c and 6a–c). Since the system is active only during summers^51^, we determine migration rates based on the amount of summers between surveys, rather than exact time.

We also observe crescent shaped bedforms in the channel. We differentiate between these bedforms and knickpoints based on scale and shape. The crescent shaped bedforms are smaller (1–5 m high), and have a more consistent wavelength (50–100 m) than the knickpoints. The bedforms have a rounded crest, and an upstream-dipping stoss side. Crescent shaped bedforms can be superimposed on knickpoints. The knickpoints themselves are 5–30 m high, are spaced 1–3 km apart in knickpoint zones, and have a sharp crest.

The pattern of alternating zones of erosion and deposition is lost in the most upstream part of the channel, above 300 m water depth (Figs. 2a and 3). The knickpoints and the erosion in the knickpoint zones progressively decrease in size upstream. Very small knickpoints might occur in this upstream part of the system, but it becomes difficult to distinguish them from crescent shaped bedforms. To understand the role of knickpoints in channel evolution, we therefore focus on the well-defined knickpoints in the main three knickpoint-zones.

### Knickpoint-zone 1

We now describe each of the three main knickpoint-zones, which are numbered from 1 to 3 in a down-channel direction (Fig. 3). Knickpoint-zone 1 migrates through a pre-existing channel bend during the time covered by the surveys (Figs. 5a and 6a). The knickpoints are focussed towards the outside of the bend (Fig. 6a). The knickpoint-zone consisted of a single frontal-knickpoint that was around 20 m high in March 2008 (Figs. 4a and 5a). A second knickpoint developed in the knickpoint-zone by February 2015 (Figs. 4a, 5a and 6a). Both knickpoints are about 10 m high from February 2015 onwards. The frontal-knickpoint migrated ~2.5 km upstream between March 2008 and October 2016, averaging at 280 m/year. The knickpoint migration has caused up to 20 m erosion of the channel floor.

### Knickpoint-zone 2

Knickpoint-zone 2 migrated through a relatively wide segment of the channel (Figs. 5b and 6b). The frontal knickpoint is 5 m high and migrated with an average rate of around 300 m/year over the entire survey, with the fastest rates of 440 m/year occurring between 2010 and 2015. The main knickpoint is 25 m high and migrated through the outside of a pre-existing channel bend. After 2010, migration of this main knickpoint completely reshaped the channel morphology, creating a new narrower and more sinuous channel. The thalweg in one of the new bends migrated partly outside the original channel (Figs. 2b, 5b and 6b). Part of the original channel bacame a terrace after knickpoint migration. The main knickpoint is smaller (around 15 m) and less active after February 2015.

### Knickpoint-zone 3

Knickpoint-zone 3 migrated through an area where the channel was not well developed (Figs. 2e, 5c and 6c). The height (around 15 m) of the frontal-knickpoint remains near-constant through the study period. Migration of the frontal-knickpoint involved erosion into previously deposited (before 2008) sediments, creating a 20 m deep and well-defined channel in locations where the channel was previously much shallower (10 m). The frontal-knickpoint migrated 1.8 km upstream during the 2008–2016 period, at a rate of around 200 m/year. A second large (around 30 m), but less-steep knickpoint can be recognised in 2008 and 2010, whilst two smaller (around 15 m high) knickpoints follow the frontal-knickpoint from 2015 onwards.

### Outer-bend erosion

Outer-bend erosion resulting in lateral migration of the channel is common in Bute Inlet, causing channels to migrate laterally up to 120 m over the entire length of the survey (Figs. 2a, c, d, g and 5d). While some progressive outer-bend erosion is observed in locations unaffected by knickpoint migration (Fig. 2d), outer-bend erosion is enhanced strongly where it is coincident with knickpoint migration (Fig. 2f).

### Crescent shaped bedforms

Crescent shaped bedforms are not easily resolvable in the deeper part of the system, due to the vertical resolution of the multibeam surveys. However, the prodeltas are dominanted by crescent shaped bedforms, and do not experience knickpoint migration. Changes in seabed elevation (<10 m) associated with crescent shaped bedform migration here are less than changes (of up to 25 m) associated with knickpoint migration (Fig. 4d).

### Levee development

Levees are a distinct feature in many submarine channels, where levee crests may rise over 100 m above the surrounding seafloor^1,54^. The levees in Bute Inlet are up to 10 m high, but typically <5 m high (Supplementary Fig. 2e, f). Channels here have a negative relief compared to the surrounding floor of the fjord, rather than bound by levees. No significant levee aggradation is recorded during the time of the survey.

### Eroded volumes

Difference maps were used to calculate volumes of erosion. We compared the total erosion in the channel, erosion caused by knickpoint migration, and outer-bend eroded sediment independent from knickpoint migration. The total amount of erosion in 9 years over the entire length of the active channel is 39 × 10^6^ m^3^. Of that total eroded volume, 28 × 10^6^ m^3^ can be attributed to knickpoint migration, which is 72% of the total eroded volume, and similar to the amount of sediment delivered into the system^47^. Outer-bend erosion accounts for 8 × 10^6^ m^3^ (21%) of the total eroded volume, and about 30% of the amount of sediment delivered to the system (Supplementary Fig. 3).

### Testing previous models for channel evolution

Our first aim is to understand what controls submarine channel evolution. It has previously been suggested that secondary (across-channel) helical flow causing gradual bend migration, is the main control on submarine channel evolution, as is the case for many rivers. There has been considerable debate over whether the sense of submarine secondary circulation is river-like or reversed^25,31,55,56^. Outer-bend erosion causing lateral migration is common in Bute Inlet and can locally reach rates of over 10 m/year. This is fast, even compared to rapidly migrating meandering rivers^30^, and almost an order of magnitude higher than the incision rate. However, our study shows that outer-bend migration can often be linked to knickpoint migration (Fig. 2g), rather than occurring gradually, as observed in rivers. This knickpoint-related lateral migration may explain the punctuated migration inferred from submarine channel deposits^57^. However, we do not observe substantial sediment deposition at inner-bends. Furthermore, long stretches of the channel in Bute Inlet are straight (around Fig. 2e), and not characterised by expanding meander bends an resulting cut-offs, as seen as in some other submarine channels^26,58^. It appears that meander bend cut-offs are not a major control on channel evolution in Bute Inlet, as none are observed in our surveys, nor are any signs of previous cut-offs observed. Secondary flow therefore does not always play the key role in submarine channel evolution, irrespective of the sense of that secondary flow compared to rivers.

Secondly, previous work has suggested that deposition of levees plays a key role in fixing channels in place, and creating flow-confinement, and thus channel evolution^33^. However, the exact role of levees in channel initiation and evolution remains a topic of debate^16,21,33,59^. Levee development may be especially important in highly depositional channels, such as channels on the Amazon Fan and Bengal Fan^1,54^. If this process is important in Bute Inlet, it acts on longer timescales, since we do not see significant deposition on the levees. However, we do see new confinement being formed independent of levees through the migration of knickpoints. These knickpoints can create a well-developed channel, where no clear channel existed previously (Figs. 5c and 6c). Similar processes have been shown in flume tank experiments where new channels were initiated by upstream-migrating erosional features^60,61^. However, such fast-moving knickpoints were only monitored once in this detail previously at field scale^46^. Furthermore, the channel in Bute Inlet confines flows by being incised in the seafloor rather than through deposition of levees rising above the seafloor.

Finally, pervasive crescent shaped bedforms on the delta-front are most likely a record of cyclic steps in supercritical turbidity currents, as similar-scale bedfoms have been linked to cyclic steps in supercritical flows at nearby Squamish Delta^17,39^. These bedforms can be an important control on submarine channel evolution in other systems^18,38^. However, we show that knickpoints play a more dominant role in Bute Inlet channel. We later discuss whether the knickpoints themselves are a supercritical flow bedform, albeit at a larger scale.

### Rapid knickpoint migration can dominate channel evolution

Here we show for the first time that fast-moving knickpoints can dominate the evolution of a submarine channel. Upstream-migrating knickpoints in Bute Inlet are fast-moving (100–450 m/year). This is 2–6 orders of magnitude faster than typical knickpoint migration rates in rivers, which is 0.001–1 m/year, depending on substrate strength and discharge^42^. However, knickpoints in rivers can occasionally migrate up to 1000 m/year due to flash-floods or weak substrate^42–44^. Migration rate of knickpoints has only been documented in three other subaqueous channels^46,62,63^, but in all cases they migrate upstream at fast rates of 50–600 m/year, comparable to those seen in Bute Inlet. Flume tank experiments of knickpoints previously suggested fast migration rates of knickpoints^61^, however direct comparison of erosion rates between experiments and natural systems remains difficult, due to scaling issues inherent in experiments. The migration rate of these knickpoints is also very high compared to other large-scale bedforms, such as tidal bars and aeolian dunes, that migrate up to 10 s of m/year^64,65^. Submarine knickpoints can also cause lateral migration of a channel thalweg, or incise new channel sections in places where no well-defined channel was previously present (Figs. 5b,c and 6b, c).

Rapid sediment deposition occurs in channel reaches between knickpoint-zones. These deposits most likely represent downstream accumulation of sediment eroded by the upstream knickpoint, as can occur in rivers^45^. However, the volume of sediment deposited downstream of the knickpoints appears to be smaller than the eroded volume upstream (Figs. 2a and 3). This difference could be due to part of the initially eroded knickpoint sediment being transported further downstream, and deposited on the distal lobe.

Volumetric estimates of surface change also demonstrate the dominance of knickpoints. Within the channel, the volume of sediment eroded by upstream-migrating knickpoints accounts for ~72% of the total observed erosion, equalling the volume of sediment supplied by the main river at the top of the channel during the same period. Even though erosion related to knickpoint migration appears to exceed the deposition during the survey period, knickpoints migrated during erosion into recently deposited channel-filling sediments (Figs. 2b and 4a). This re-incision into recent deposits can explain why migration of many individual 5–30 m deep knickpoints, over periods of centuries to millennia, has not carved a deeper channel along this fjord. Phases of erosion caused by upstream-migrating knickpoints, followed by phases of deposition, appear to create a balance such that the channel depth is approximately that of a single knickpoint (5–30 m).

Reworking of recently deposited, and thus poorly consolidated sediment could partly explain why knickpoint migration is so rapid. Fresh channel deposits are mostly sand-dominated^48,50^, and they may be prone to erosion and failure, especially when loaded or scoured by fast moving turbidity currents. This kind of substrate may be much weaker than older, and consolidated or cemented sediments, or bedrock, which underlies many river systems.

### How do knickpoints migrate?

**Fig. 7:**
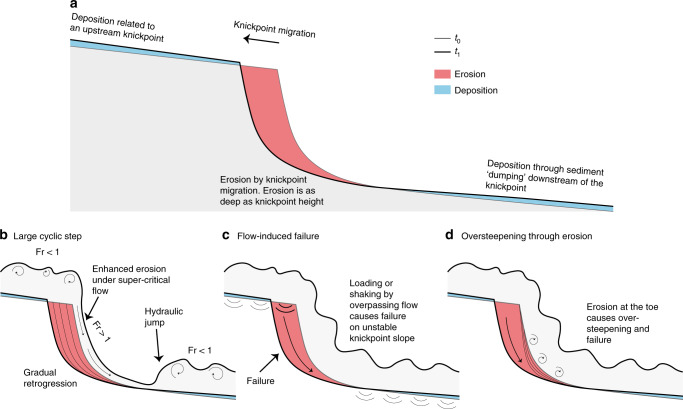
Contrasting models for knickpoint migration. **a** Generalised pattern of erosion and deposition associated with upstream-migration of knickpoints. **b** Cyclic step model. Knickpoint is formed by repeated instabilities (termed cyclic steps) that are self-generated by supercritical turbidity currents going through a hydraulic jump. **c** Flow-induced slope-failure model. Knickpoint results from sudden failure of the channel floor, when loaded during passage of a turbidity current. **d** Oversteepening through undercutting in which erosion at the toe of the steep face causes oversteepening, and eventual failure.

Knickpoints migrate upstream along the channel, so we infer that their migration is caused by turbidity currents which are common in Bute Inlet^49–51^. We propose three internal flow-substrate processes that could trigger knickpoint migration, either in isolation or in combination (Fig. 7). The first model is that submarine knickpoints, and intervening areas of deposition, are a large-scale bedform produced and maintained by instabilities within supercritical flow^35,66–68^, but with far longer wavelengths (>1–5 km) than those of the crescent shaped bedforms (typically 50–100 m in Bute Inlet; Fig. 4d). The second model is that migrating knickpoints are formed by seabed failures triggered by rapid undrained loading of the substrate, as a turbidity current passes. Unusually rapid rates of sediment accumulation (up to 1 m/year) in the depositional areas of the channel floor may favour such failure^36,69^. Past work suggested this model to explain the migration of sub-lacustrine knickpoints in tailing deposits^70^. These studies show that failure and subsequent knickpoint migration can even occur unrelated to an overpassing turbidity current. Third, the base of knickpoints may be gradually eroded and undercut by turbidity currents, leading to oversteepening and failure^20^. This process is similar to headwall undercutting described in waterfalls and is known to cause migration of knickpoints in rivers, albeit at much slower rates^43,71^.

We conclude that all three models are potentially consistent with our field data. It is thus uncertain which model is correct, and more detailed monitoring will be needed to discriminate between competing hypotheses with confidence.

### How are submarine knickpoints created or destroyed?

These three models (Fig. 7) explain the movement of existing knickpoints, rather than their origin or final disappearance. We consistently observe three knickpoint-zones in our time-lapse surveys (Figs. 3 and 4a–c). Some additional knickpoints appear within these zones, but they may be due to break-up of a larger knickpoint (Fig. 4a–c). Thus, we do not record a clear example of a new knickpoint-zone forming, though we can speculate on their creation. Knickpoints are common in river systems, where they are often related to external factors, including local tectonic movement, variability in substrate or bedrock strength, or base-level change^41^. Similar external controls have been suggested for submarine knickpoints^20,72^.

However, none of the knickpoints in Bute Inlet can be related to any of these external factors. There is no evidence of local active tectonics, based on seismographs that locate earthquakes. The submarine knickpoints are carved mainly into recently deposited channel-fill sediment^48,50^ (Figs. 2a and 4a), making a strong bedrock or substrate control unlikely. As the channel is underwater, changes in sea-level (base-level) will not produce knickpoints. Furthermore, these submarine knickpoints are not created by meander-bend cut-offs, as observed in rivers, and modelled for submarine channels^73^. There are no meander bend cut-offs or remnants of meander bend cut-offs along the Bute Inlet submarine channel (Figs. 1 and 2a).

The lowermost knickpoint in knickpoint-zone 3 was in 2008 only 5–10 km away from the channel to lobe transition zone (where channel confinement ends and sediment deposits in a lobe^74^). A migration rate of 200 m/year in knickpoint-zone 3 would suggest this knickpoint was at the channel-to-lobe transition zone around 1958–1983. We would expect to see signs of some such external controls, if those created knickpoints in the recent past. Therefore, it appears that knickpoints can be created internally in submarine channels. If we rule out that knickpoints are created far beyond the downstream end of the system, we suggest that knickpoints are created by internal dynamics around the channel-to-lobe transition zone. A small steep step in channel gradient can be observed around this area, which may eventually form the next knickpoint zone (Fig. 3a).

The exact origin of these knickpoint zones thus remains unclear at present. Similarly, we do not see the disappearance of knickpoint zones as they migrate up-channel over the 9 years of our surveys. Further observations are thus also needed to establish how knickpoints are born and disappear, potentially through even longer sequences of repeat surveys.

### Implications for evolution of submarine channel-bends

**Fig. 8:**
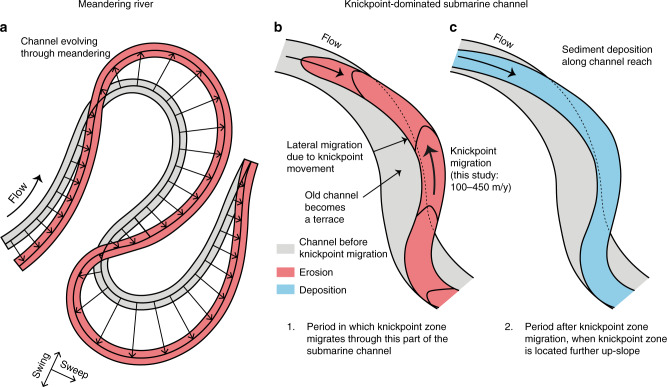
Comparison between migration of channel bends in meandering rivers and submarine channels dominated by fast-moving knickpoint zones. **a** Outer-bank erosion leads to swing and sweep of bends in a meandering river (after^30^). **b** Rapid knickpoint zone migration in a submarine channel leads to incision, lateral migration, and terrace formation. **c**) Knickpoint zone migrates further up-slope, and this part of the submarine channel is then infilled by deposition. Deposited sediment is partly sourced from knickpoint erosion occuring further up-slope.

We now seek to understand how knickpoint migration affects the evolution of submarine channel bends. The planform evolution of meandering river bends is dominated by secondary (across-channel) helical flow, which causes point-bar deposition on the inner-bend, and erosion of the outer-bend^27^ (Fig. 8a). This in turn causes river meander bends to progressively increase in amplitude (swing) and translate downstream (sweep)^30,75,76^ (Fig. 8a). A recent review found that submarine channel bends evolve in different ways, depending on what kind of bend-related (often bank attached) bars form^5^. These bars are controlled by patterns of near-bed secondary flow, or direct suspended load fallout. This would result in submarine channel evolution being driven by deposition in bend-related (often bank-attached) bar deposits.

However, here we observe that submarine channel-bend evolution is dominated by rapid knickpoint migration, causing sudden channel-wide erosion (Fig. 2). Rapid sediment deposition then occurs in channel-reaches downstream from knickpoint-zones (Fig. 2a, e), rather than formation of distinct bend-related bars. Our surveys also show that migration of knickpoints can extend outside the original channel, and thus create terraces (Figs. 5b and 6b). This, combined with the lack of meander bend cut-offs or gradually migrating bends, produces a rather different view of evolution of channel-bends than previously described^5,23^ (Fig. 8b).

### Implications for submarine channel deposits

**Fig. 9:**
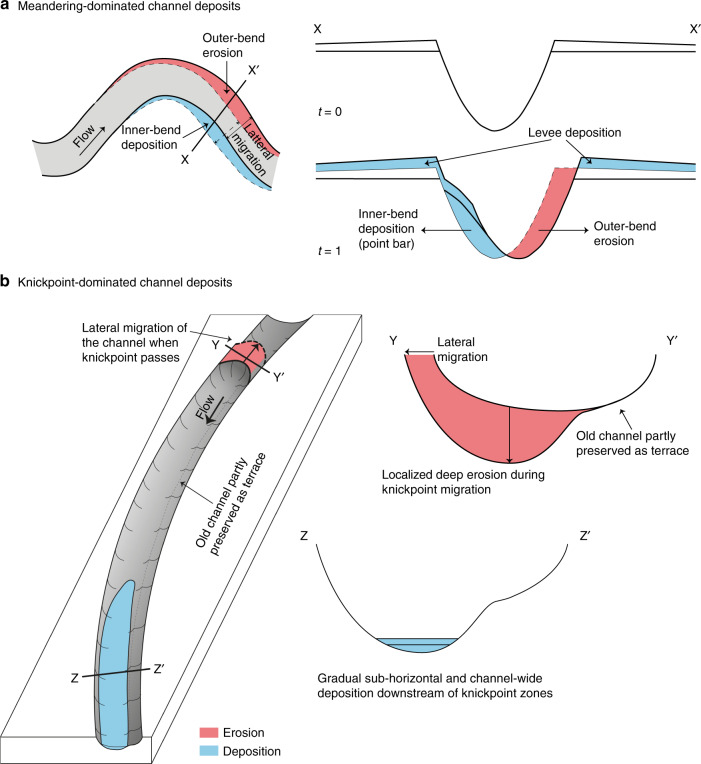
Generalized models for submarine channel evolution and deposits. **a** Deposition and erosion in meandering dominated channels (after^23^). This results in bars (shown in light blue) deposited in the inner bends, and erosion in the outer bends. The erosion causes outward and downstream propagation of bends. **b** New model for submarine channel deposits in locations dominated by fast-moving knickpoints, such as Bute Inlet. Knickpoint migration causes deep erosion, and potential channel migration. This is then followed by channel-wide deposition, once the knickpoint has migrated further upslope.

Knickpoint migration can also have a profound impact on the detailed architecture of channel-fill deposits (Fig. 9). Knickpoint migration is mainly associated with erosion into and reworking of previous sandy deposits within the channel-fill (Fig. 2b). Sediment is deposited gradually (~1 m/year) downstream of knickpoints in channel-wide sheets extending several kilometres along the channel (Figs. 2a, b and 9b). These patterns of deposition and erosion due to knickpoints are fundamentally different to the bend-related bars predicted previously, based on more gradual bend-migration driven by secondary across-channel flow^5,23^ (Fig. 9a).

Submarine channels can be subdivided according to whether they are net-erosional, net-depositional, or there is a balance between erosion and deposition over longer (100 s to 1000 years) periods^77^. Channels formed by long-term net-erosion, often termed submarine canyons, may contain only thin deposits with limited preservation potential. In contrast, areas of net-deposition will tend to produce systems confined by levees raised high above the surrounding seafloor^1^, and they will have better potential for preservation in the rock record. Bute Inlet appears to represent an intermediate situation, in which erosion and deposition along the submarine channel are nearly balanced. Thus, over longer time scales, the knickpoint deposits in such settings will not be fully preserved as they are formed here; they will be mostly reworked by successive knickpoint erosion and deposition. Only if the channel reaches a net-depositional stage or moves laterally, parts of these deposits might be preserved.

### Similar knickpoints occur in other locations worldwide

Various types and dimensions of seabed knickpoints have been documented in numerous locations worldwide^20,72^ (Supplementary Table 2). These locations include knickpoints with broadly similar dimensions that occur in active submarine and sub-lacustrine channel systems. Knickpoints in other systems are often linked to tectonics, bedrock outcrop or meander-bend cut-off^20,73^. However, similar knickpoints are found in Monterey Canyon, South China Sea, Capbreton Canyon, and others, where a clear external trigger is also lacking^22,46,66^. The type of knickpoints seen in Bute Inlet and other locations, can occur in a wide range of systems, including locations with low (<1°) gradients. Furthermore, erosional features that share similarities with knickpoints have been reported to migrate up the channels in Squamish Delta^18^. This suggests that the processes that form fast-moving channel-knickpoints, and their impacts on submarine channel evolution and deposits, might be of widespread importance.

### A new generalised model for submarine channels

We use 9 years of time-lapse bathymetry from an active submarine channel in Bute Inlet, British Columbia, to study how submarine channels evolve. Rapid (100–450 m/year) upstream-migration of knickpoints was the dominant process driving channel evolution. Previously described processes such as meander-bend migration, levee aggradation, and migration of smaller bedforms all play a minor role in channel evolution on this time scale in Bute Inlet. Knickpoints are steep (up to angle of repose) steps in channel gradient, with heights of up to 30 m. Sediment upstream of a knickpoint is eroded during migration and deposition occurs further downstream of the knickpoint. Deposits form long and thin channel-wide deposits, rather than previously proposed bend-related bars. Knickpoints can migrate outside the banks of the original channel, causing lateral migration of the channel and development of channel bends. Previous models proposed outer-bend erosion and inner-bend deposition due to across-channel (secondary) flow, as the main control on evolution of channel bends and their resulting deposition. However, here we propose an alternative model for submarine channel evolution and deposits, controlled by upstream-migrating knickpoints. Finally, as similar knickpoints occur in sedimentary channels in a variety of subaqueous settings worldwide, we suggest the processes described here are common globally.

## Methods

### Multibeam bathymetry acquisition

This study uses five bathymetric surveys spanning a total of 9 years, collected in March 2008, November 2010, February 2015, June 2016, and October 2016. Past work has considered only the first two surveys in 2008 and 2010^52,53^. The survey in March 2008 survey was obtained using a Kongsberg-Simrad EM 1002 (100 kHz) multibeam echosounder. The later surveys used a Kongsberg Maritime EM710 (70–100 kHz) multibeam echosounder, controlled using Kongsberg Maritime SIS software. Data were processed to correct for differences in sound velocity of the water (using data from a sound velocity profiler), together with tides, waves, and ship’s motion. The vertical resolution of bathymetric data is ~0.5% of the water depth, and is thus a maximum of ~3 m at the channel termination at water depths of ~600 m (Supplementary Fig. 2b, c, d). Bathymetry was then processed to calculate the local gradient, in order to optimally display small steep topographic features such as knickpoints.

### Difference maps

Patterns of erosion and deposition are visualised using bathymetric difference maps, calculated by subtracting two surveys from each other. These difference maps were then used to estimate volumes of different erosional processes. First, the total eroded volume within the active channel is calculated (Supplementary Fig. 3). Then, parts of that eroded volume are attributed to either outer-bend erosion or knickpoint migration, based on the geometry and location of erosional areas (Supplementary Fig. 3). Steep areas such as fjord sidewalls and the overbanks have not been taken into account, because volumetric calculations including these areas will reflect uncertainties rather than real change. Reliable volumetric calculations and mass balances of the deposition cannot be made, as the thin and widespread geometry of depositional bodies often falls below resolution of the surveys, especially on the overbanks.

### Channel profiles

The bathymetric surveys were used to construct along-channel profiles. The position of the channel shifts as the channel evolves, so profiles were constructed along the position of the thalweg in that survey. The different along-channel profiles were all normalised to before comparing.

## Supplementary information

Supplementary Material

## Data Availability

The data that support the findings of this study are available from the corresponding author upon reasonable request.
