# Author Correction: Rapidly-migrating and internally-generated knickpoints can control submarine channel evolution

**DOI:** 10.1038/s41467-020-18394-9

**Published:** 2020-09-01

**Authors:** Maarten S. Heijnen, Michael A. Clare, Matthieu J. B. Cartigny, Peter J. Talling, Sophie Hage, D. Gwyn Lintern, Cooper Stacey, Daniel R. Parsons, Stephen M. Simmons, Ye Chen, Esther J. Sumner, Justin K. Dix, John E. Hughes Clarke

**Affiliations:** 1 National Oceanography Centre, European Way, Southampton, SO14 3ZH UK; 2 Ocean and Earth Sciences, National Oceanography Centre, University of Southampton, European Way, Southampton, SO14 3ZH UK; 3 Departments of Geography and Earth Sciences, University of Durham, South Road, Durham, DH1 3LE UK; 4 Natural Resources Canada, Geological Survey of Canada, Box 6000, 9860 West Saanich Road, Sidney, BC Canada; 5 Energy and Environment Institute, University of Hull, Cottingham Road, Hull, HU6 7RX UK; 6 Earth Sciences, Center for Coastal & Ocean Mapping, University of New Hampshire, 24 Colovos Road, Durham, NC USA; 7 Present Address: Department of Geoscience, University of Calgary, T2N 1N4 Calgary, Alberta Canada

Correction to: *Nature Communications* 10.1038/s41467-020-16861-x, published online 19 June 2020.

The original version of this Article contained an error in the labelling of the cross-section in Fig. 2g and the vertical axis in Fig. 2b.

This has been corrected in both the PDF and HTML versions of the Article.

