# Supplementary material for: Rapidly-migrating and internally-generated knickpoints can control submarine channel evolution
Source: Nat Commun. 2020 Jun 19;11:3129. doi: 10.1038/s41467-020-16861-x (PMC7305132; doi:10.1038/s41467-020-16861-x)
Supplement: Supplementary file 1 — Supplementary Material [file 41467_2020_16861_MOESM1_ESM.pdf]

Supplementary Material for:

Rapidly-migrating and internally generated knickpoints can control submarine channel evolution

by Heijnen et al.

Supplementary Figures

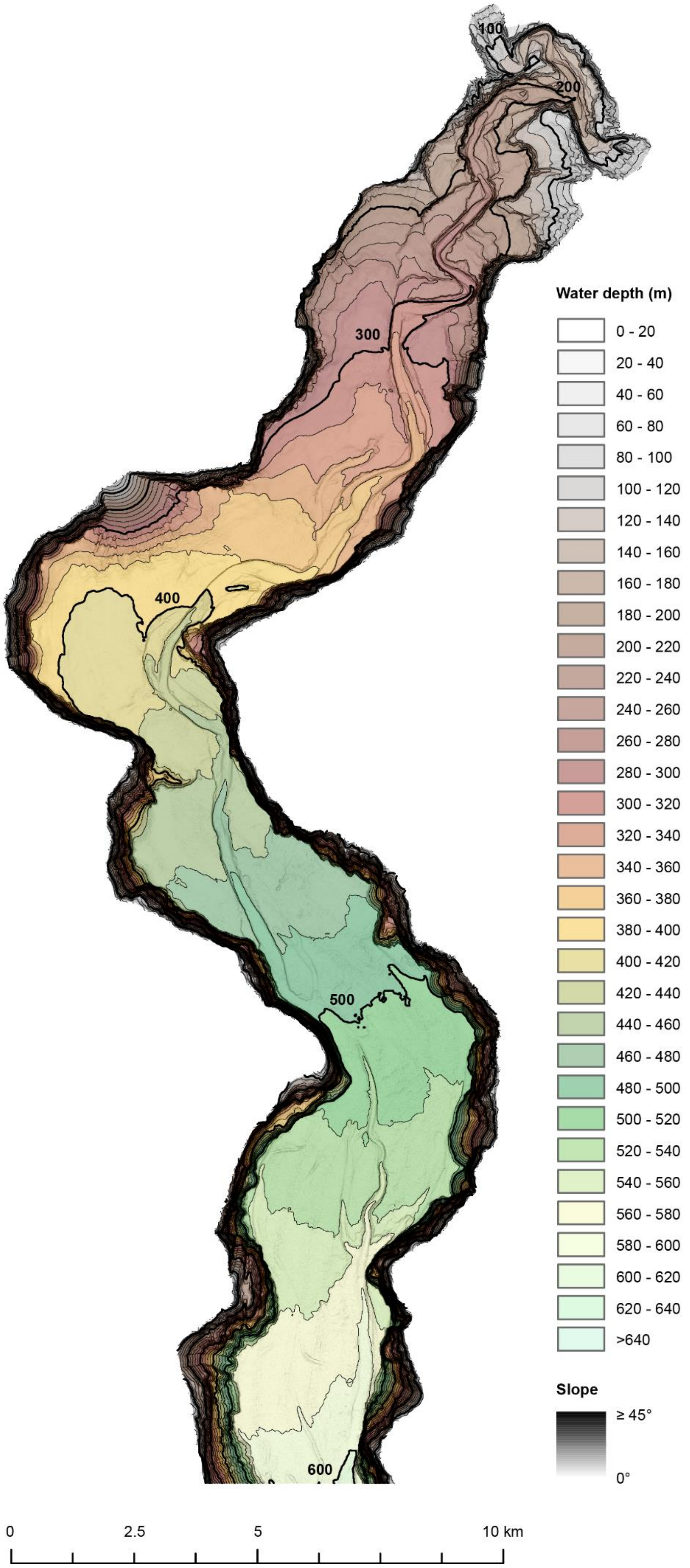

Supplementary figure 1: Bathymetric map of Bute Inlet classified in 20 m intervals.

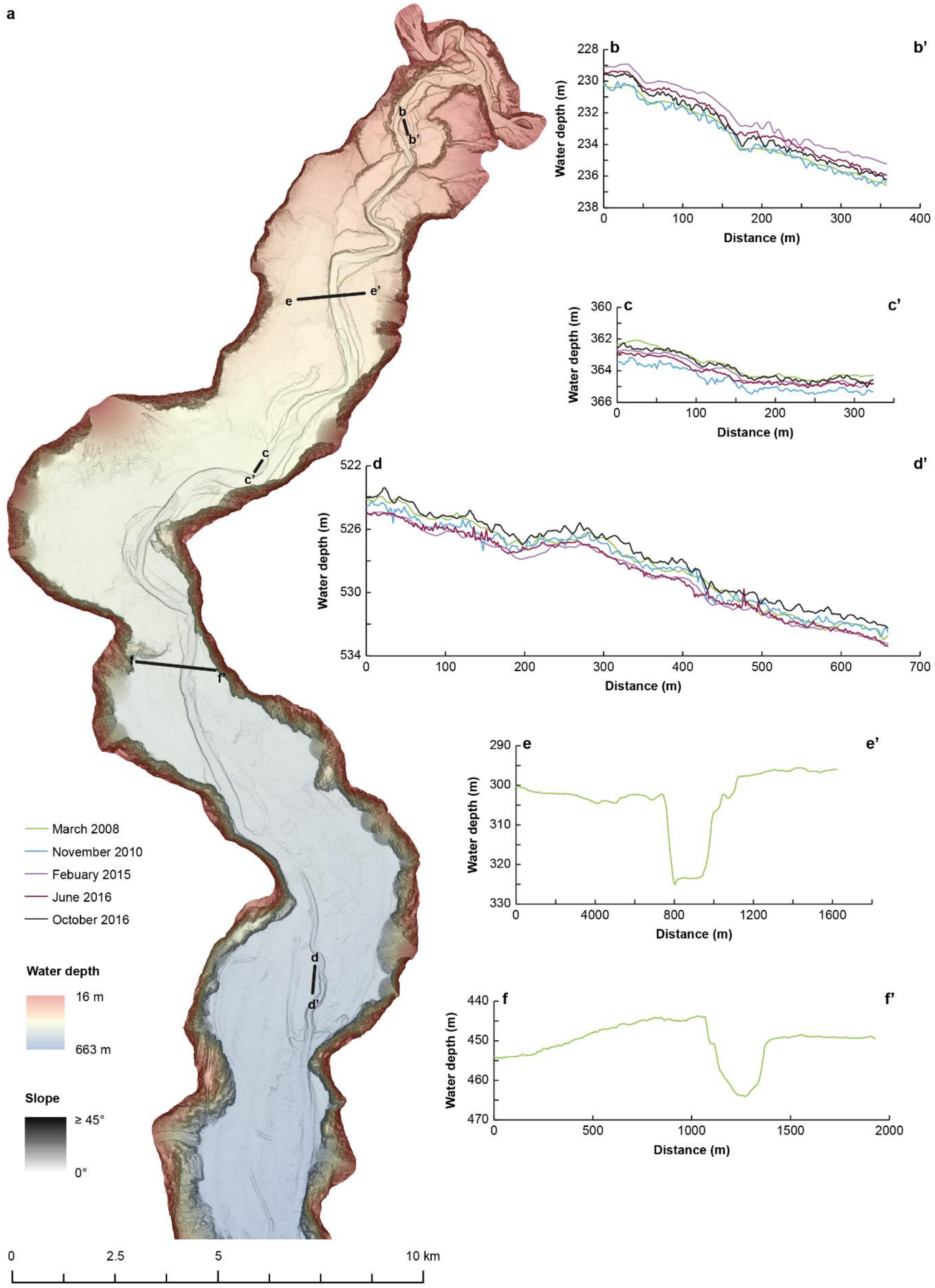

Supplementary figure 2: Profiles along the seafloor of Bute Inlet in 2008 demonstrating vertical resolution and the system's levees. a) location of cross sections. b-d) profiles of areas that are assumed to not be subject to seafloor change. The variability within the different surveys is up to ~0.5% of the water depth. e) profile through the channel, showing that levees can be completely absent and the system is insiced in the surrounding seafloor. f) profile through channel, showing a particular well developed levee of about 10 m high.

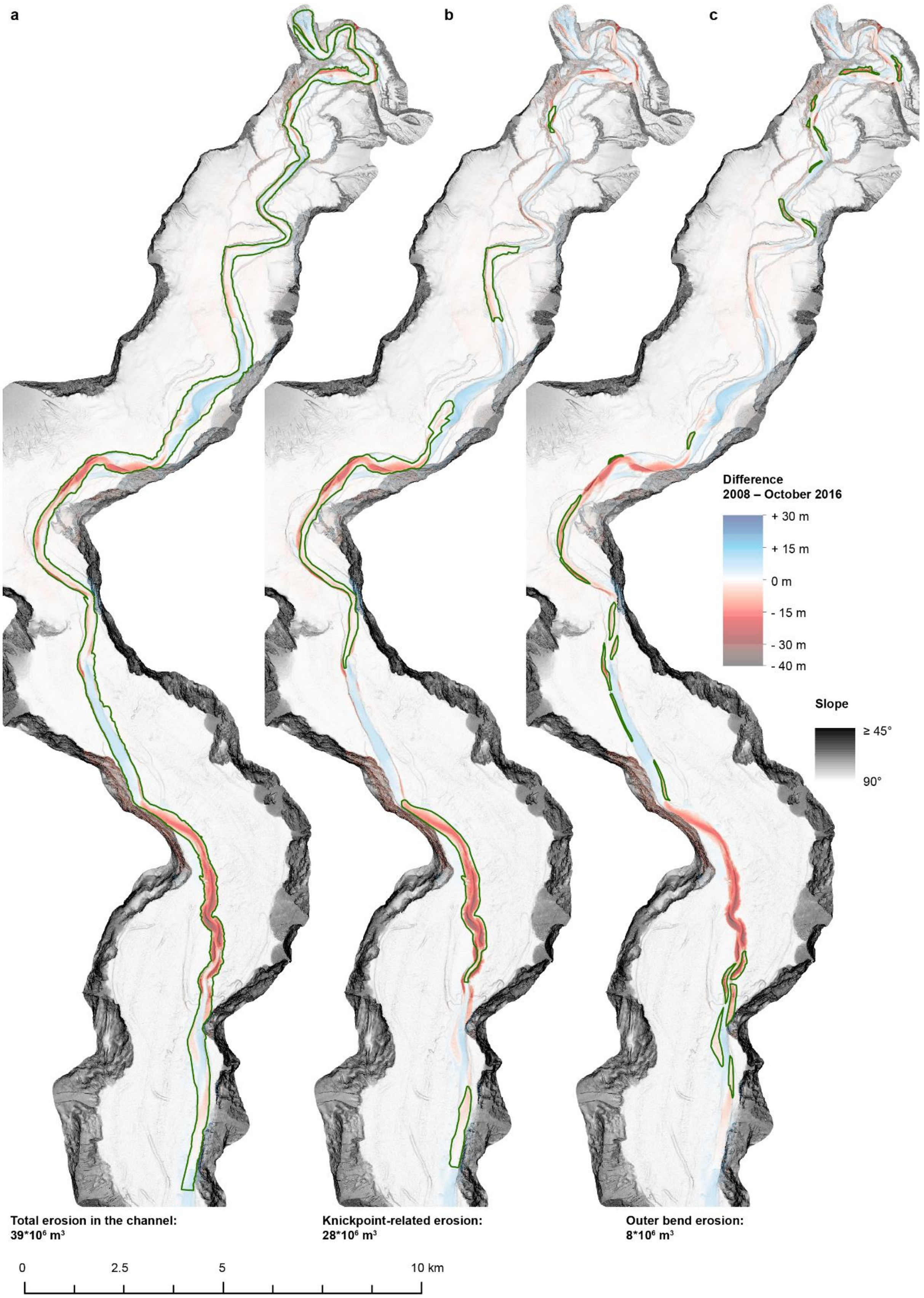

Supplementary figure 3: Overview of areas selected (outlined in green) for volumetric estimates of a) the total erosion in the channel, b) Knickpoint-related erosion, and c) Outer-bend erosion.

Supplementary Tables

Supplementary Table 1: Time lapse bathymetric surveys of subaqueous channel systems		
Location	Key Reference(s)	Notes
Fraser Delta, BC, Canada	[1]	Channels on a delta slope. Up to 300 m water depth. 8 surveys between 1994 and 2006. Channel is occasionally dredged. Kickpoints (referred to as erosional scarps or schallop shaped depression) are seen. Several other different bedforms are observed.
Bute Inlet, Knight Inlet and Toba Inlet, BC, Canada	[2]	Fjords. Submarine channels fed by fjord head deltas. Up to 660 m waterdepth. 2 surveys per fjord between 2005 and 2010 years. Includes this study area. Knickpoint and associated erosion in Bute Inlet is visible in the figures.
Squamish Delta, BC, Canada	[3–9]	Fjord. Recently reset system fed by a fjord head delta. Up to 200 m water depth. Single beam and sidescan surveys from 1974 onwards. 9 multibeam surveys between 2004 and 2009; 93 surveys in 2011, including daily during the summer of 2011. Sub-daily surveys in the summer of 2013. 6 daily surveys in the summer of 2015.
Monterey Canyon, California, west coast USA	[10–12]	Marine. Canyon on continental slope fed by littoral cells. 7 surveys of the upper 4 km of the canyon between 2002 and 2005. 3 more surveys in 2007 using an AUV. 2 surveys cover the system up to 2100m water depth, one in 2002 and a big AUV effort obtained in 2008-2009. 6 more AUV surveys in specific locations between 2015 and 2017. Crescentic shaped bedforms and knickpoints are observed.
Capbreton Canyon, Bay of Biscay, France	[13,14]	Marine. Canyon on continental slope. Up to 3500 m water depth. Focus on the canyon head (up to 400 m water depth). 10 surveys between 1998 and 2018. Crescentic shaped bedforms and knickpoints are abundant.
Lake Geneva, Switzerland/France	[15]	Lacustrine. Up to 300 m waterdepth. Surveys in 2000 and 2012. Contains knickpoint and crescentic shaped bedforms.
Malalay Canyon, Mindoro Island, the Philippines	[16]	Marine. Canyon in continental shelf. Up to 350 m water depth. 26 surveys between 1997 and 2018, but variable coverage. Canyon-wide surveys at least in 2007, 2008, 2011, and 2018. Crescentic shaped bedforms are common.
Pearl River Mouth Basin, South China Sea	[17]	Marine, canyons in upper continental slope. 500-1700 m water depth. One survey in 2004/2005 and one in 2018
Wabush Lake, NL, Canada	[18]	Lacustrine, system created by tailings. Up to 100 m water depth. Surveys in 1999, 2004, 2006, and 2008. Knickpoints are common.
Pointe Odden, Gabon	[19]	Marine, several smaller channels in a bay fed by a littoral cell. Up to 75 m water depth 6 surveys between 2004 and 2009. Channel incision and a landslide.
Ligurian Margin (incl. Var canyon), Mediterranean, France	[20]	Marine. Several channels/canyons on an active margin. Fed by rivers. Single beam surveys from 1960s and 1970s. Multibeam surveys from 1991, 1999, 2006, and 2011. Focus on landslide history. Erosional areas in several channels can be distinguished.
Kaikoura Canyon, New Zealand	[21]	Marine. Canyon on a continental slope. Over 2000 m water depth. 2 surveys, one before and one after the 2016 earthquake. Focus on earthquake triggered mass movement.
Stromboli, Southern Messina Strait and Punta Alice, Italy	[22]	Marine, canyon on active margin. Up to 400 m water depth. Two surveys between 2005 and 2007. Focus on terrestrial debris flows triggering hyperpycnal flows.

Begwan Solo delta, Java, Indonesia	[23]	Pro-delta channels on continental shelf. Up to 30 m water depth. Surveys in 2008 and 2012.
Westerschelde, Netherlands	[24]	Estuary. Up to 25 m water depth. Dredging induced failure of the side of the main estuary channel.
Lower St. Lawrence Estuary, Eastern Canada	[25,26]	Inner continental shelf. Sediment starved canyons. Up to 325 m water depth. seven surveys between 2007 and 2017. Upstream-migrating crescentic shaped bedforms.
Eastern Baffin Island, north-eastern Canada	[27,28]	Mapped 31 fjord-head deltas between 2006 and 2014. Repeat bathymetry is available for several of these fjords.

Supplementary Table 2: Subaqueous knickpoints in literature

Location	Setting	Comments	Key Reference(s)
Wabush Lake, NL, Canada	Lacustrine. Iron tailings dumping; 10 km long, up to 100 water depth.	Around 10 main knickpoints. Typical 1–2 m high and 10–30 m wide (up to 4 m high and 70 m wide)	[18,29]
Offshore New Jersey, east coast USA.	Continental slope, up to 2200 m water depth.	Eight knickpoints, typically a few tens of meters high. One major knickpoint of 200 m high. Knickpoints probably formed due to differences in lithology	[30]
Gulf of Alaska, USA	Accretionary prism, up to 4500 m water depth.	13 knickpoints, typically 50–150 m high, but up to 350 m high. Knickpoints have a tectonic origin (localised vertical movement; anticlines), however fault related knickpoints cannot be ruled out in some cases.	[30]
Astoria Canyon, offshore Oregon, west coast USA	Canyon on accretionary wedge. Up to 2300 m water depth.	Six tectonically controlled knickpoints, tens of meters high.	[30]
San Antonio Canyon, offshore Chile	Canyon in forearc basin. Up to 5500 m water depth.	A single 500-1000 m high knickpoint. The shape may suggest a landslide origin, rather than usual erosion by turbidity currents. Also a 50 m high knickpoint.	[30]
Southern Barbedos Accretionary Prism, Caribbean.	Canyons through fold ridges on an accretionary prism. Up to 3500 m water depth.	Knickpoints related to tectonic structures, several hundreds of meters high.	[30,31]
Niger Delta, offshore Niger	Continental slope. Up to 3400 m water depth. Reconstructed using seismic surfaces.	Five knickpoints, or knickpoint zones of tens of meters high per zone. Related to a fault and thrust belt and diapirsim.	[32,33]
Espirito Santo Basin, offshore Brasil	Continental slope influenced by salt tectonics. Reconstructed using seismic surfaces	Five 18-30 m high knickpoints recognized below present-day seabed depressions.	[34]
North Sardinia Slope, Mediterranean	Passive margin with three main channels reaching up to 1000 m water depth.	Several smaller (around 10 m) knickpoints and two big (around 50 m) knickpoints.	[35]
Monterey Canyon, offshore California, west coast USA	Continental slope, up to 3600 m water depth.	5-10 m high knickpoints are common in the upper (up to 1300 m water depth) canyon	[36,37]

		Five up to 200 m high steep steps outside main channel, referred to as discontinuous scours. Interpreted to be cyclic steps associated with channel inception.	
La Jolla Canyon, offshore California, west coast USA.	Continental slope. Up to 700 m water depth.	Seven up to 30 m high knickpoints referred to as 'distinctive canyon floor scarps' in the axial channel.	[38]
Lake Geneva, Switzerland.	Lacustrine. Up to 300 m water depth.	Upstream migrating incision, and steep steps in channel profile. Additionally, knickpoint-bound headless channels are present	[15]
South China sea	Continental slope. Several canyons/channels. Up to 3500 m water depth.	Around 40 10-81 m high steps and several smaller steps in two canyons. Steps are interpreted as cyclic step bedforms.	[39]
Knight, Toba, and Bute Inlets, BC, Canada	Fjords. Up to 650 m water depth	16 up to 40 m high knickpoints in channels in three different fjords.	[40]
Santa Monica and Redondo Canyon, California, west coast USA	Continental slope. Up to 800 m water depth.	Six up to 70 m high knickpoints and three 'distinctive canyon floor scarps'. Large scours (up to 70 m high) are found outside the channel too.	[41]
Amazon fan, offshore Brazil	Continental slope. Up to 3700 m water depth. Reconstructed using seismic surfaces.	Single large knickpoint related to channel avulsion.	[42]
East Breaks, Gulf of Mexico	Salt-withdrawal mini-basins.	Fault related knickpoints.	[42]
Rhone Fan, offshore France, Mediterranean	Active margin. Up to 2500 m water depth.	Several knickpoints related to channel avulsion.	[42]
Congo Canyon, offshore Angola/Republic of the Congo/ Democratic Republic of the Congo	Continental slope. Up to 5000 m water depth.	Several knickpoints recognised in the channel-levee part of the system and are linked to avulsions.	[43]
Magdalena Channel, offshore Columbia	Active margin. Up to 3500 m waterdepth.	Knickpoints are common and associated with avulsions.	[44]
Madeira channel system, Southwest of Madeira, Atlantic Ocean	Intra basins channel system on passive margin. 4400–5400 m water depth.	Knickpoints bound upstream sides of headless channels. Suggested that knicpoints are the channel initiators	[45]
Tenryu Canyon, offshore Japan	Active margin canyon, up to 4000 m water depth.	One steep fault related knickpoint.	[46]
Offshore Angola	Channel surface of an ancient channel system on a passive margin. Reconstructed using seismic surfaces.	Avulsion related knickpoints in order of tens of meters high.	[47]

Danube Canyon, Black Sea	Canyon-channel system up to 1000 m water depth.	One knickpoint, marking the shift between the canyon and channel regime.	[48]
Central Atlantic USA margin	Canyons on passive continental margin. Up to ~1800 m water depth.	Knickpoints are observed and attributed to variability in substrate erodability.	[49]
Capbreton Canyon, Bay of Biscay, France	Canyon in continental slope. Up to 3500 m water depth. Focus on the canyon head up to 400 m water depth.	Up to 80 potential knickpoints present. Up to 7 m high. Migrate with 90-600 m/yr	[14]

Supplementary References

1. Hill, P. R. Changes in Submarine Channel Morphology and Slope Sedimentation Patterns from Repeat Multibeam Surveys in the Fraser River Delta, Western Canada. in *Sediments, Morphology and Sedimentary Processes on Continental Shelves* 47–69 (2013).
2. Conway, K. W., Barrie, J. V., Picard, K. & Bornhold, B. D. Submarine channel evolution: Active channels in fjords, British Columbia, Canada. *Geo-Marine Lett.* **32**, 301–312 (2012).
3. Hughes Clarke, J. E., Brucker, S., Hill, P. & Conway, K. Monitoring morphological evolution of fjord deltas in temperate and Arctic regions. in *Rendiconti Online Societa Geologica Italiana* **7**, 147–150 (2009).
4. Hughes Clarke, J. E. *et al.* Temporal progression and spatial extent of mass wasting events on the Squamish prodelta slope. in *Landslides and Engineered Slopes: Protecting Society through Improved Understanding - Proceedings of the 11th International and 2nd North American Symposium on Landslides and Engineered Slopes, 2012* 1091–1096 (2012).
5. Hughes Clarke, J. E., Vidiera Marques, C. R. & Pratomo, D. Imaging active mass-wasting and sediment flows on a Fjord Delta, Squamish, British Columbia. in *Submarine Mass Movements and Their Consequences, 6th International Symposium* 249–260 (2016).
6. Hughes Clarke, J. E. First wide-angle view of channelized turbidity currents links migrating cyclic steps to flow characteristics. *Nat. Commun.* **7**, 1–13 (2016).
7. Hizzett, J. L. *et al.* Which Triggers Produce the Most Erosive, Frequent, and Longest Runout Turbidity Currents on Deltas? *Geophys. Res. Lett.* **45**, 855–863 (2018).
8. Hage, S. *et al.* How to recognize crescentic bedforms formed by supercritical turbidity currents in the geologic record: Insights from active submarine channels. *Geology* **46**, 563–566 (2018).
9. Vendettuoli, D. *et al.* Daily bathymetric surveys document how stratigraphy is built and its extreme incompleteness in submarine channels. *Earth Planet. Sci. Lett.* **515**, 231–247 (2019).
10. Smith, D. P., Ruiz, G., Kvitek, R. & Iampietro, P. J. Semiannual patterns of erosion and deposition in upper Monterey Canyon from serial multibeam bathymetry. *Geol. Soc. Am. Bull.* **117**, 1123 (2005).
11. Smith, D. P., Kvitek, R., Iampietro, P. J. & Wong, K. Twenty-nine months of geomorphic change in upper Monterey Canyon (2002-2005). *Mar. Geol.* **236**, 79–94 (2007).
12. Paull, C. K. *et al.* Origins of large crescent-shaped bedforms within the axial channel of Monterey Canyon, Offshore California. *Geosphere* **6**, 755–774 (2010).
13. Mazières, A. *et al.* High-resolution morphobathymetric analysis and evolution of Capbreton submarine canyon head (Southeast Bay of Biscay-French Atlantic Coast) over the last decade using descriptive and numerical modeling. *Mar. Geol.* **351**, 1–12 (2014).
14. Guiastrennec-Faugas, L. *et al.* Upstream migrating knickpoints and related sedimentary processes in a submarine canyon from a rare 20-year morphobathymetric time-lapse (Capbreton submarine canyon, Bay of

Biscay, France). *Mar. Geol.* **423**, 106143 (2020).

15. Corella, J. P. *et al.* The role of mass-transport deposits and turbidites in shaping modern lacustrine deepwater channels. *Mar. Pet. Geol.* **77**, 515–525 (2016).

16. Sequeiros, O. E. *et al.* How typhoons trigger turbidity currents in submarine canyons. *Sci. Rep.* **9**, (2019).

17. Yin, S. *et al.* Continental slope-confined canyons in the Pearl River Mouth Basin in the South China Sea dominated by erosion, 2004–2018. *Geomorphology* **344**, 60–74 (2019).

18. Turmel, D., Locat, J. & Parker, G. Morphological evolution of a well-constrained, subaerial-subaqueous source to sink system: Wabush Lake. *Sedimentology* **62**, 1636–1664 (2015).

19. Biscara, L. *et al.* Submarine slide initiation and evolution offshore Pointe Odden, Gabon — Analysis from annual bathymetric data (2004–2009). *Mar. Geol.* **299**–**302**, 43–50 (2012).

20. Kelner, M. *et al.* Frequency and triggering of small-scale submarine landslides on decadal timescales: Analysis of 4D bathymetric data from the continental slope offshore Nice (France). *Mar. Geol.* **379**, 281–297 (2016).

21. Mountjoy, J. J. *et al.* Earthquakes drive large-scale submarine canyon development and sediment supply to deep-ocean basins. *Sci. Adv.* **4**, (2018).

22. Casalbore, D., Chiocci, F. L., Scarascia Mugnozza, G., Tommasi, P. & Sposato, A. Flash-flood hyperpycnal flows generating shallow-water landslides at Fiumara mouths in Western Messina Strait (Italy). *Mar. Geophys. Res.* **32**, 257–271 (2011).

23. Syahnur, Y. Geomatics Best Practices in Saka Indonesia Pangkah Limited (Case Study: Ujung Pangkah Pipeline Integrity). in *Indonesian Petroleum Association* (2018).

24. Mastbergen, D. *et al.* Multiple flow slide experiment in the Westerschelde estuary, The Netherlands. in *Advances in Natural and Technological Hazards Research* **41**, 241–249 (2016).

25. Normandeau, A. *et al.* Morphodynamics in sediment-starved inner-shelf submarine canyons (Lower St. Lawrence Estuary, Eastern Canada). *Mar. Geol.* **357**, 243–255 (2014).

26. Normandeau, A. *et al.* Retreat Pattern of Glaciers Controls the Occurrence of Turbidity Currents on High-Latitude Fjord Deltas (Eastern Baffin Island). *J. Geophys. Res. Earth Surf.* **124**, 1559–1571 (2019).

27. Hughes-Clarke, J. E. *et al.* Reconnaissance seabed mapping around Hall and Cumberland peninsulas , Nunavut : opening up southeastern Baffin Island to nearshore geological investigations. *Summ. Act. 2014* 133–144 (2015).

28. Normandeau, A. *et al.* Storm-induced turbidity currents on a sediment-starved shelf: Insight from direct monitoring and repeat seabed mapping of upslope migrating bedforms. *Sedimentology* **67**, 1045–1068 (2020).

29. Turmel, D. *et al.* Morphodynamic and Slope Instability Observations at Wabush Lake, Labrador. *Submar. Mass Movements Their Consequences* **28**, 435–446 (2010).

30. Mitchell, N. C. Morphologies of knickpoints in submarine canyons. *Bull. Geol. Soc. Am.* **118**, 589–605 (2006).

31. Huyghe, P., Foata, M., Deville, E., Mascle, G. & Group, C. W. Channel profiles through the active thrust front of the southern Barbados prism. *Geology* **32**, 429–432 (2004).

32. Adeogba, A. A., McHargue, T. R. & Graham, S. A. Transient fan architecture and depositional controls from near-surface 3-D seismic data, Niger Delta continental slope. *Am. Assoc. Pet. Geol. Bull.* **89**, 627–643 (2005).

33. Heiniö, P. & Davies, R. J. Knickpoint migration in submarine channels in response to fold growth, western Niger Delta. *Mar. Pet. Geol.* **24**, 434–449 (2007).

34. Heiniö, P. & Davies, R. J. Trails of depressions and sediment waves along submarine channels on the continental margin of Espirito Santo Basin, Brazil. *Bull. Geol. Soc. Am.* **121**, 698–711 (2009).

35. Dalla Valle, G. & Gamberi, F. Slope channel formation, evolution and backfilling in a wide shelf, passive

continental margin (Northeastern Sardinia slope, Central Tyrrhenian Sea). *Mar. Geol.* **286**, 95–105 (2011).

36. Fildani, A., Normark, W. R., Kostic, S. & Parker, G. Channel formation by flow stripping: Large-scale scour features along the Monterey East Channel and their relation to sediment waves. *Sedimentology* **53**, 1265–1287 (2006).

37. Paull, C. K., Caress, D. W., Ussler, W., Lundsten, E. & Meiner-Johnson, M. High-resolution bathymetry of the axial channels within Monterey and Soquel submarine canyons, offshore central California. *Geosphere* **7**, 1077–1101 (2011).

38. Paull, C. K. *et al.* Anatomy of the La Jolla Submarine Canyon system; offshore southern California. *Mar. Geol.* **335**, 16–34 (2013).

39. Zhong, G., Cartigny, M. J. B., Kuang, Z. & Wang, L. Cyclic steps along the South Taiwan Shoal and West Penghu submarine canyons on the northeastern continental slope of the South China Sea. *Geol. Soc. Am. Bull.* **127**, 804–824 (2015).

40. Gales, J. A. *et al.* What controls submarine channel development and the morphology of deltas entering deep-water fjords? *Earth Surf. Process. Landforms* **44**, 535–551 (2019).

41. Tubau, X. *et al.* Submarine canyons of Santa Monica Bay, Southern California: Variability in morphology and sedimentary processes. *Mar. Geol.* **365**, 61–79 (2015).

42. Pirmez, C., Beaubouef, R. T., Friedmann, S. J. & Mohrig, D. C. Equilibrium Profile and Baselevel in Submarine Channels: Examples from Late Pleistocene Systems and Implications for the Architecture of Deepwater Reservoirs. in *Deep-Water Reservoirs of the World: 20th Annual* 782–805 (2000).

43. Babonneau, N., Savoye, B., Cremer, M. & Klein, B. Morphology and architecture of the present canyon and channel system of the Zaire deep-sea fan. *Mar. Pet. Geol.* **19**, 445–467 (2002).

44. Estrada, F., Ercilla, G. & Alonso, B. Quantitative study of a Magdalena submarine channel (Caribbean Sea): implications for sedimentary dynamics. *Mar. Pet. Geol.* **22**, 623–635 (2005).

45. Stevenson, C. J. *et al.* The flows that left no trace: Very large-volume turbidity currents that bypassed sediment through submarine channels without eroding the sea floor. *Mar. Pet. Geol.* **41**, 186–205 (2013).

46. Soh, W. & Tokuyama, H. Rejuvenation of submarine canyon associated with ridge subduction, Tenryu Canyon, off Tokai, central Japan. *Mar. Geol.* **187**, 203–220 (2002).

47. Gee, M. J. R. & Gawthorpe, R. L. Submarine channels controlled by salt tectonics: Examples from 3D seismic data offshore Angola. *Mar. Pet. Geol.* **23**, 443–458 (2006).

48. Popescu, I. *et al.* The Danube submarine canyon (Black Sea): morphology and sedimentary processes. *Mar. Geol.* **206**, 249–265 (2004).

49. Mitchell, N. C. Form of submarine erosion from confluences in Atlantic USA continental slope Canyons. *Am. J. Sci.* **304**, 590–611 (2004).
